# Microsatellite instability and manifestations of angiogenesis in stage IV of sporadic colorectal carcinoma

**DOI:** 10.1097/MD.0000000000013956

**Published:** 2019-01-04

**Authors:** Włodzimierz Otto, Finlay Macrae, Janusz Sierdziński, Justyna Smaga, Maria Król, Ewa Wilińska, Krzysztof Zieniewicz

**Affiliations:** a Department of General, Transplant & Liver Surgery; b Department of Medical Informatics & Telemedicine; c Department of Oncology, Hematology & Internal Medicine; d Department of Pathology Central Teaching Hospital, Medical University of Warsaw, Banacha 1a, 02-097 Warsaw, Poland; e Department of Colorectal Medicine and Genetics, The Royal Melbourne Hospital, and Department of Medicine, The University of Melbourne, Australia.

**Keywords:** angiogenesis, colorectal carcinoma, liver metastases, microsatellite instability

## Abstract

Angiogenesis represents one of the critical mechanisms that facilitates carcinoma development. The study objective was to evaluate whether the microsatellite instability of colorectal carcinoma has impact on the angiogenesis activity in liver metastases.

In a cohort of 80 randomly selected patients with stage IV colorectal carcinoma, 30% were recognized as microsatellite unstable (Microsatellite instability high-frequency (MSI-H)). The endothelial progenitor cell fraction (CD309+) was counted within the subpopulation of CD34+CD45+ cell and CD34+CD45- cells by flow cytometer. vascular endothelial growth factor (VEGF) factor levels were quantified in serum samples by enzyme-linked immunosorbent assay (ELISA). A control group consisted of 36 healthy volunteers. The relationship of genomic instability to angiogenesis activity was evaluated by multivariate analysis in comparison to the controls, adopting a *P* < .05 value as statistically significant.

The expression of endothelial progenitor cells (EPCs) and VEGF was significantly higher in MSI-H compared to both microsatellite stability (MSS) patients and healthy controls (*P* < .008). Multi-parametric analysis showed microsatellite instability (OR=9.12, *P* < .01), metastases in both lobes (OR = 32.83, *P* < .001) and simultaneous metastases outside liver (OR = 8.32, *P* < .01), as independent factors associated with increased angiogenesis as assessed by measures of EPC and VEGF. A higher percentage of EPCs within the white blood cell fraction (total % EPCs / white blood cells (WBC)) and higher serum concentrations of VEGF were present in patients with MSI-H colorectal cancer, and not with MSS cancers (*P* < .001).

MSI-H patients with colorectal cancer metastases are associated with the overexpression of circulating EPCs and VEGF, potentially driving angiogenesis. This should be considered in therapeutic decision-making.

## 1 Introduction

Metastases to the liver from colorectal carcinoma are the clinical manifestation of stage IV of the disease. Surgery and complementary chemotherapy with several drugs used usually in combination with 5-fluorouracil is actual a chance for cure for these patients with long-term survival in case of successful resection. Unfortunately, the results are unpredictable and new metastases appear quickly, despite radical eradication of the primary lesions.^[1–4]^

Angiogenesis is a mechanism important for colorectal carcinoma development and progression.^[5–7]^ Several signaling pathways contribute to such activity as stimulators acting in a synergistic manner with the Vascular Endothelial Growth Factor (VEGF) and the Vascular Endothelial Growth Factor Receptor (VEGFR) pathways.^[8–10]^ VEGF is a potent angiogenesis agent that acts as a specific mitogen for vascular endothelial cells through specific cell surface receptors. Both VEGF and its receptor are expressed at high levels in metastatic human colon carcinomas and in tumor associated endothelial cells, and production of these two proteins correlates directly with the degree of tumor vascularization.^[11–13]^ A prevalent hypothesis is that the cells that make up the new lining of blood vessels which respond to the tumor cytokines, belong to the primitive blasts of both hematopoietic and endothelial origin (EPCs). They can be recognized in the circulation by the positive reaction with the cluster differentiated antigen CD309 (KDR) within subpopulation of CD34+, CD133+, CD45+ cells of hematopoietic origin, and subpopulation of CD34+, CD133+, CD45- cells of endothelial origin. The flow cytometer and the ISHAGE protocol is validated to be as a simple, rapid, and sensitive method of quantification of the both these subpopulations among the white blood cells (WBC). The standardization is based on the use of state-of-the-art bright fluorochrome conjugates and the combination of the CD34, CD45, CD133, and CD309 markers.^[14–23]^

Quantitative changes in the circulating Endothelial Progenitor Cells (EPCs) population, therefore, might be considered as a significant indicator of angiogenesis activity.^[24–26]^

The heterogeneous nature of colorectal carcinoma and the changes in their genomic integrity also play a considerable role in the generation of drug resistance and in mechanisms promoting development of metastases to the liver.^[27–29]^ Most sporadic colorectal carcinomas are microsatellite stable; microsatellite instability (MSI) applies to a small percentage of patients, consistently about 15%. Several trials have demonstrated better relapse-free survival and overall survival, as well as decreased risk of metastases for MSI tumors, compared with patients with MSS tumors.^[30–33]^ Several reports have described also the relationship between MSI status and worse response to chemotherapy based on 5-fluorouracil.^[34–36]^ The guidelines of European Society for Medical Oncology suggest that MSI should be evaluated to guide the selection of chemotherapeutic agents to treat colorectal cancer.^[37,38]^

The current study objective is to evaluate any association of genetic instability with angiogenesis activity in patients with MSI-H and MSS CRCs treated surgically for liver metastases.

## 2 Methods

**Figure 1:**
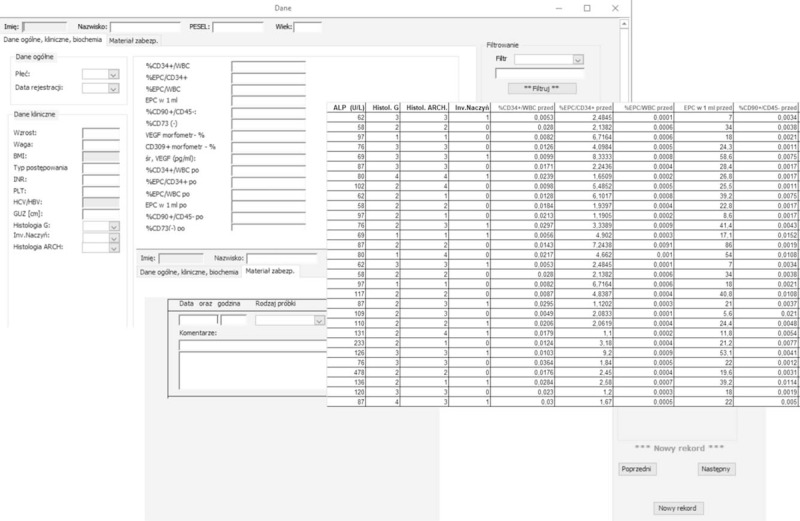
An example of several forms of the telemedical system designed to establish interrelations between an increase in the number of endothelial progenitor cells and other factors of angiogenesis (VEFGF).

The telemedical system designed to establish interactions between the increase in the number of endothelial progenitor cells and other angiogenesis factors (VEFGF) and genetic instability of the DNA mismatch repair (MMR) tumor in the metastasis of colorectal cancer to the liver in terms of practical clinical staging assessment was prepared for the research project, predicting treatment outcomes and assessing the risk of relapse after a radical excision of metastatic lesions. The developed WEB application was written using the Java language and uses only the open Free and Open Source tool standards. Figure 1 illustrates several forms of this system along with a section of the database.

## 3 Patients

The study covered a cohort of 80 randomly selected patients of both genders, in the age range of 35 to 80 years (mean 54.1 years). They were admitted to the Department of General, Transplant & Liver Surgery, Medical University of Warsaw, for operative treatment of colorectal liver metastases. All patients had undergone surgical treatment for a colorectal carcinoma. At the time of the primary tumor resection, they were in clinical stage I-IV according to the American Joint Committee on Cancer (AJCC) classification. Those who underwent colorectal resection for clinical stage I carcinoma at their primary assessment later underwent a course of chemotherapy that started at the onset of metastases; those in clinical stage II – III later underwent chemotherapy as adjuvant chemotherapy following the resection of their primary cancer; those in stage IV underwent a course of chemotherapy to complement the initial surgery to manage their disease. The presence of metastatic tumors was confirmed in all patients by ultrasound examination (USG), computed tomography (CT), and magnetic resonance imaging (MRI). After standard pre-operative preparation, patients underwent either local tumor excision or partial liver resection for metastatic liver tumors as it was required.

Patients were categorized by the expression of the MLH1 and MSH2 mismatch repair enzymes within the metastatic liver tumor tissue because the loss of MLH1 and MSH2 very close associates with MSI. Those who did not express at least one of the MMR enzymes were classified to mismatch instability (MSI) group. The groups were matched in relation to demographic characteristics, tumor nodes metastases classification (TNM) stage of disease, tumor localization and the type of resection done for their primary cancer. Out of 24 patients with metastatic liver disease from MSI-H group, 16 received standard systemic mono- or multi- drug fluorouracil-based chemotherapy in different combinations, 4 received the anti-EGF drug (Cetuximab) and 4 were treated with the anti-VEGF drug (Avastin) alone or in combinations; of 56 patients from MSS group, 43 received standard systemic mono- or multi- drug fluorouracil-based chemotherapy in different combinations, 7 received the anti-EGF drug (Cetuximab) and 6 were treated with anti-VEGF drug (Avastin) alone or in combinations. The details are given in Table 1.

**Table 1:**
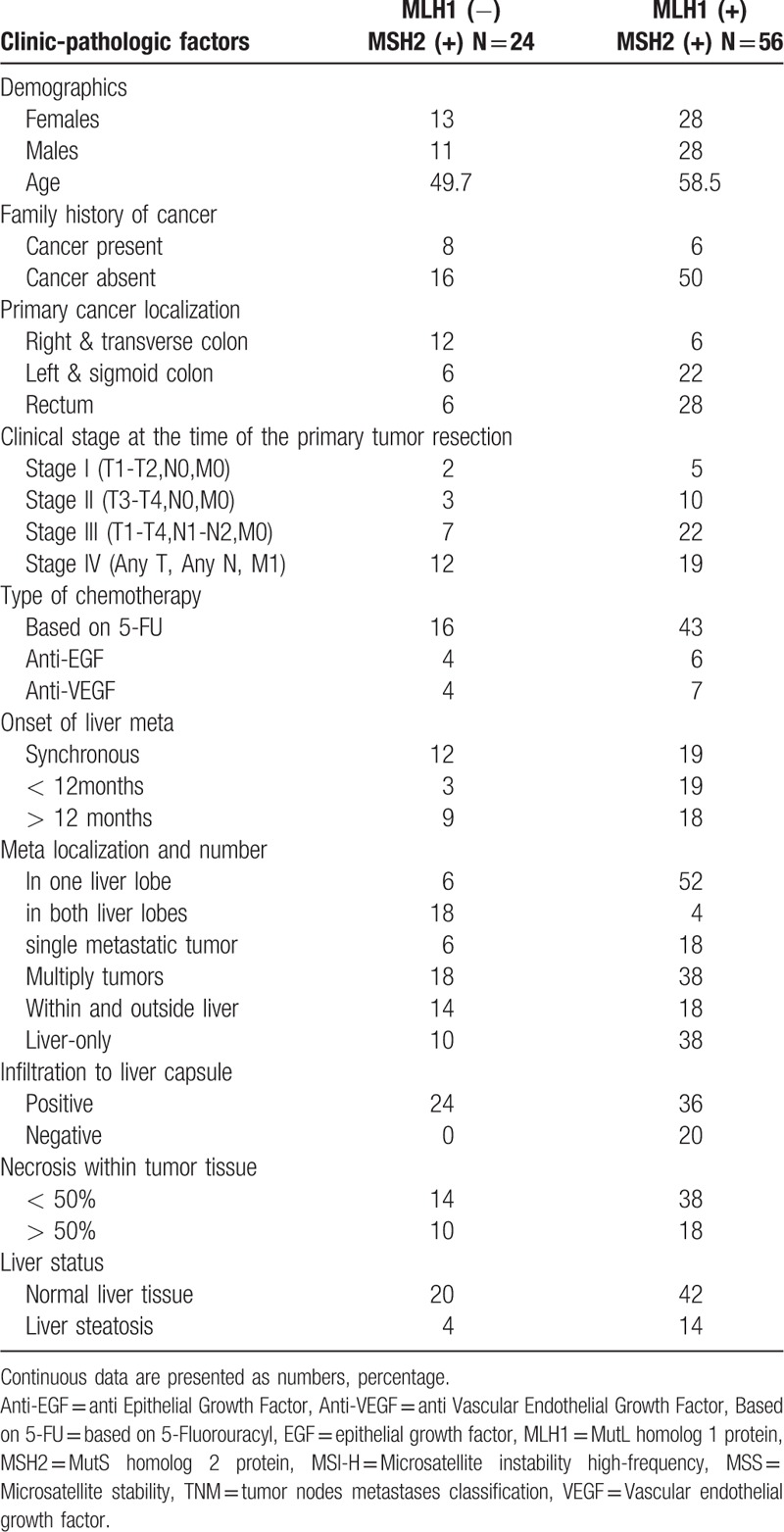
Clinic-pathologic characteristic of MSI and MSS patients with liver metastases of sporadic colorectal carcinoma.

Surgical resection specimen analysis and tumor MLH1/MSH2 protein expression surgical specimens were examined by an expert pathologist. They were fixed in 10% formalin and embedded in paraffin.

**Figure 2:**
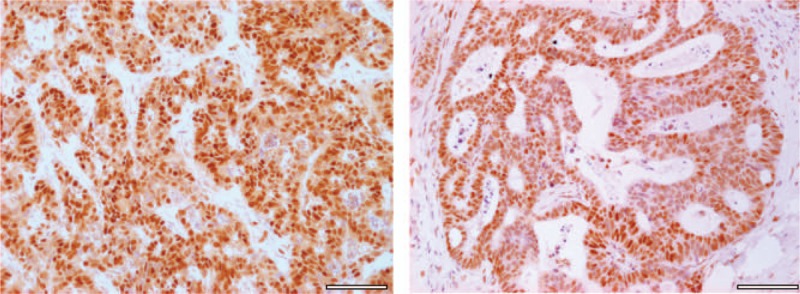
The examples of positive staining with anti-MLH1 and anti-MSH2 antibody within the tissue of the colorectal liver metastatic tumor. A. Positive staining for MLH1 protein B. Positive staining for MSH2 protein. MLH1 = MutL homolog 1 protein, MSH2 = MutS homolog 2 protein.

Multiple 4 μm-thick sections were cut from blocks and stained with hematoxylin and eosin to study the features of the metastatic tumor, and the tumor surrounding liver tissue. Tissue sections were immunohistochemically stained with the rabbit monoclonal anti-MLH1 and rabbit polyclonal anti-MSH2 antibodies (Abcam). One block of formalin-fixed, paraffin embedded tumor tissue was selected per case. The procedure included de-paraffinizing the block, followed by high-temperature antigen retrieval and the incubation with the primary antibody in the 1:200 concentration. Chromogen 3,3’-diaminobenzidine (DakoCytomation) was applied next and the cell nuclei were counterstained with the hematoxylin. The slides were analyzed under Nikon Eclipse 80i microscope. Positive nuclear staining was detected in sections of all patients stained with the anti-MSH2 antibody and in sections from 70% of patients stained with the anti-MLH1 antibody. The examples are presented in Figure 2.

### 3.1 Assessment of angiogenesis factors

**Figure 3:**
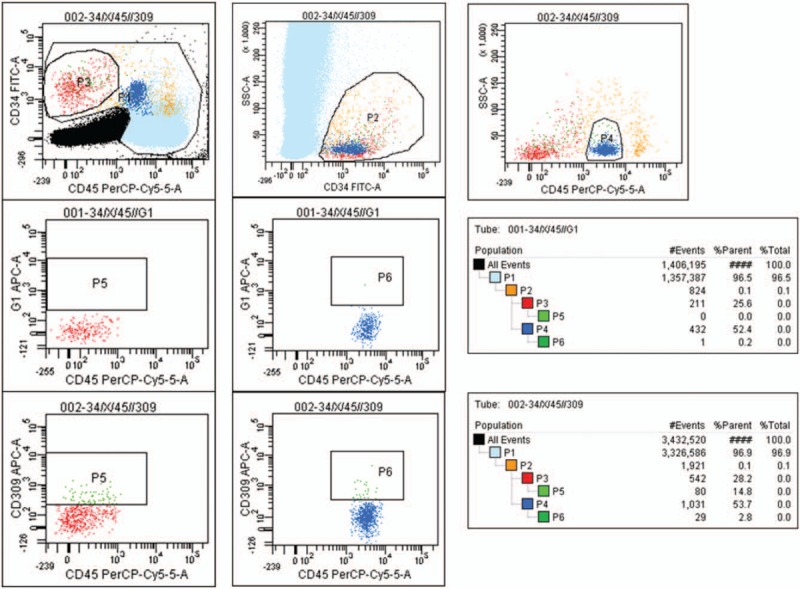
The rating of CD309+ cells in the population of CD34+CD45- and CD34+CD45+ cells of WBC in CRC patients with a positive expression of MLH1 and MSH2 mismatch repair enzyme (MSS group). The cells of CD309+ were identified in both populations in relation to the isothypic control. Pictures indicate the small number of the CD309+ cells. CRCs = colorectal carcinomas, MLH1 = MutL homolog 1 protein, MSH2 = MutS homolog 2 protein, MSS = microsatellite stability, WBC = white blood cells.

**Figure 4:**
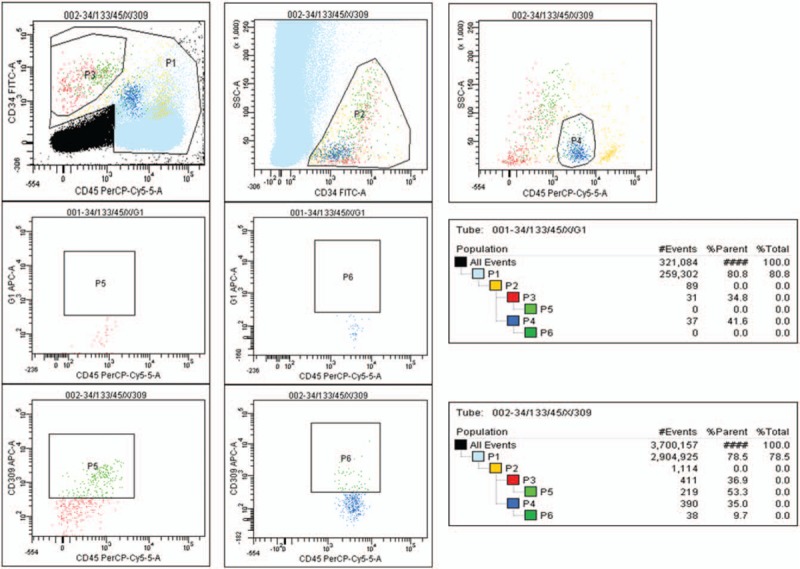
The rating of CD309+ cells in the population of CD34+Cd45- and CD34+CD45+ cells of WBC in CRC patients with the loss of expression of MLH1 mismatch repair enzyme (MSI group). The cells of CD309+ were identified in both populations in relation to the isothypic control. Pictures indicate the large number of the CD309+ cells. CRCs = colorectal carcinomas, MLH1 = MutL homolog 1 protein, WBC = white blood cells.

Two milliliters of the venous blood samples were analyzed with the flow cytometer (FACS CANTO II - BD Biosciences) for cells possessing the phenotype of CD309+ within the subpopulation of CD34+CD45+ (hematopoietic origin) and of CD34+CD45- cells (endothelial origin) within the population of the white blood cells (WBC). A gating strategy was established to separate the desired cell fractions from irrelevant cell populations, as recommended by the International Society of Hematology and Graft Engineering. The immunofluorescence of the cells for CD309 was assessed after identification of CD34 cells within the fraction of the cells positive and negative for the CD45 marker. Because it was anticipated that cells positive for CD34 and CD309 would be in very low abundance, we increased the total number of acquired events in the flow cytometer analysis to at least 2,000,000. The endothelial stem cells (EPCs45-) were defined by the phenotype CD309+CD34+CD45- and the endothelial progenitor cells (EPCs45+) were defined by the phenotype CD309+CD34+CD45+. The size of each fraction was expressed as a percentage of WBC cells. The size of each fraction of these cells was also added together and expressed as the sum of circulating endothelial cells (CECs) in proportion to WBC cells count (% CECs/WBC).^[39,40]^ The examples of the small and the large numbers of the CD309+ cells are presented in Figures 3 and 4.

VEGF levels were measured with the ELISA assay kit for measuring human serum VEGF concentration (Quantikine Human VEGF immunoassay). The sensitivity of the assay was 9 pg/ml.

### 3.2 Control group

A control group consisted of 36 healthy individuals (18 females, 18 males, mean age 38 years), admitted for operative treatment of inguinal hernia (11 pts.) or for being the living liver donors for their relatives (15 pts.) and 10 students who agreed to be the volunteers. The standard medical examination and laboratory tests have confirmed the good health status of the volunteers. They underwent tests for the same parameters and according to the same procedures as cancer patients

### 3.3 Statistical analysis

Statistical analyses were carried out using Program SAS 9.4. The quantitative data of the EPCs enumeration and the serum VEGF concentration level were expressed as median, and mean +/- SD. The univariate analysis by using the *X*^*2*^ test and also nonparametric tests (Mann-Whitney and Kruskal-Wallis) were performed to determine the interrelationships between the manifestation of angiogenesis indicators (the rate of circulating EPCs and the level of VEGF concentration), genomic instability and tumor properties. Logistic regression was performed by using features of the primary and metastatic tumors and expression of mismatch repair enzymes in the tissue of metastases. These were adopted as the independent variables, whereas quantitative data of the EPCs and VEGF were adopted as the dependent variables. A *P* value < .05 was adopted as statistically significant.

### 3.4 Ethics

The study protocol was approved by the Bioethics Committee of the Medical University of Warsaw No. KB/14/2015.

Informed consent was obtained from all cancer patients undergoing liver resection, as well as from all volunteers who were the control group.

All authors declare no conflict of interest.

## 4 Results

**Figure 5:**
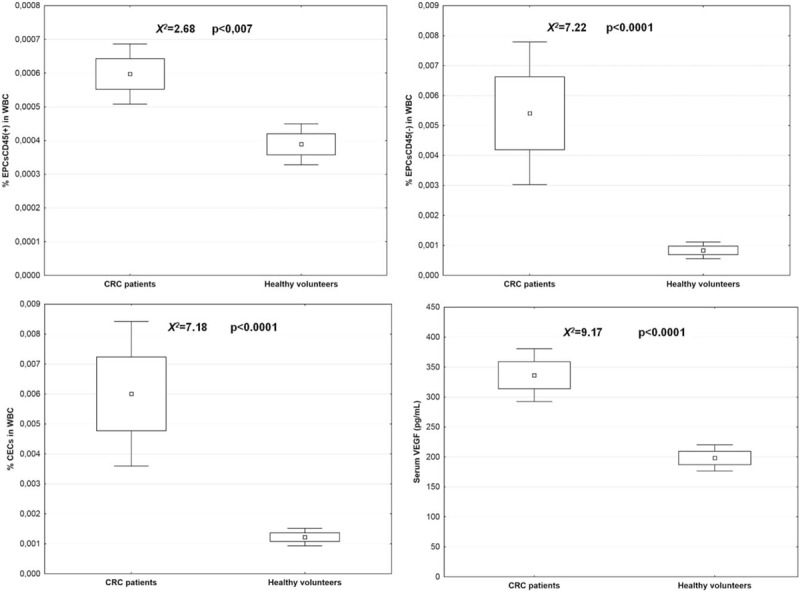
Mean values of circulating EPCs numbers and serum VEGF concentration in patients with colorectal cancer liver metastases and control group. EPCs = endothelial progenitor cells, VEGF = vascular endothelial growth factor.

Significantly higher numbers of the endothelial cells (both the EPCs45- and the EPCs45+), and higher serum levels of the VEGF protein were found in patients with CRC liver metastases in comparison to the cancer-free individuals (*X*^*2*^ = 7.18, *P* < .001 for EPCs and *X*^*2*^ = 9.17, *P* < .001 for VEGF). The EPCs/WBC ratio correlated with the serum concentration of VEGF protein in CRC patients (r = 0.53, *P* < .007 for %CECs/WBC and r = 0.62, *P* < .001 for VEGF), whereas in patients free of cancer they did not (NS). The details are given in Figure 5.

Positive nuclear staining for MLH1 and MSH2 antibodies were detected in tumor sections of 54 (70%) patients (MSS group); in 24 (30%), negative nuclear staining for MLH1 but positive for MSH2 was detected (MLH1 related MSI-H group). The distribution of cancers in the colon and rectum was similar for MSS and MSI-H cancers. However, the patients with MSI-H liver metastases differed from the patients with MSS liver metastases by the more frequent presence of the history of any cancer disease within the family (*X*^*2*^ = 25.24, *P* < .001), the presence of metastases in both liver lobes (*X*^*2*^ = 38.79, *P* < .001), synchronous metastases in and outside the liver (*X*^*2*^ = 25.24, *P* < .001), and the invasion of cancer into the liver capsule (*X*^*2*^ = 28.05, *P* < .001). On the other hand, the time between the treatment of primary tumor and the detection of metastases, the number of liver metastases and the necrotic area within the tumor, which related to adjuvant chemotherapy, were similar in patients with both MSI and MSS liver metastases. The details are given in Table 2.

**Table 2:**
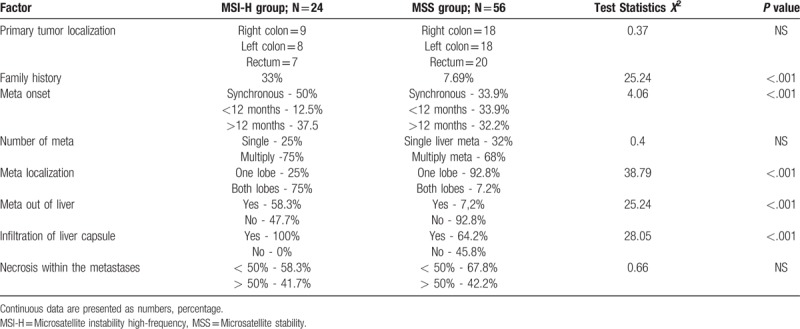
The differences in clinical and pathologic features between metastatic liver tumors in the MSI-H and MSS colorectal cancer patients.

**Figure 6:**
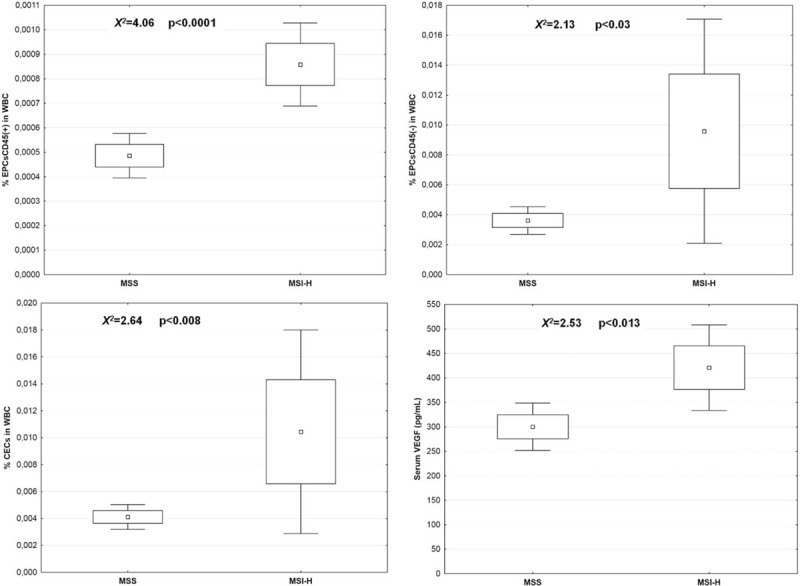
Mean values of numbers EPCs and serum VEGF concentration in patients with MSI-H and MSS colorectal cancer liver metastases. EPCs = endothelial progenitor cells, MSS = Microsatellite stability, MSI-H = Microsatellite instability high-frequency, VEGF = vascular endothelial growth factor.

The percentage of circulating endothelial cells (% CECs/WBC), as well as the percentage of both EPCs45- and EPCs45+ fractions within the white blood cell population, and the levels of serum concentration of VEGF were significantly higher in the patients with MSI-H metastases in comparison to the MSS metastases, (0.0104, SD+/−0.0189 versus 0.0041, SD+/−0.0035, *X*^*2*^ = 2.64, *P* < .008, and 420.93, SD+/−218.57 versus 300.27, SD+/−184.06, *X*^*2*^ = 2.53, *P* < .013 for VEGF). The significant correlations between % CECs/WBC, and serum concentration of VEGF protein were found only in the group of CRC patients with MSI-H metastases; (r = 0.56 *P* < .004 for %EPCs, r = 0.52, *P* < .008 for VEGF). There were no such correlations found in the MSS group. The details are given in Figure 6.

The effect of chemotherapy was different in relation to the angiogenesis factors in patients with MSI-H compared with MSS liver metastases. In the 43 MSS patients who received fluorouracil-based chemotherapy, the percentage of EPCs in WBC count and VEGF serum concentrations were lower in comparison to 13 MSS patients who received chemotherapy with anti-EGFR or anti-VEGF drugs for treatment of the metastases (U = 3.4, *P* < .001 for EPCs and U = 3.61, *P* < .001 for VEGF). On the other hand, in the 24 patients with MSI-H metastases, a significant decrease in the percentage of EPCs in WBC count (*X*^*2*^ = 11.25, *P* < .003) was noted only in 4 patients who received Avastin. The reduction effect was observed mostly in relation to number of CD309+CD45(-) stem cells of the endothelial origin and in relation to the serum concentration of VEGF protein, not to the number of CD309+CD45(+) stem cells of the hematopoiesis origin (*X*^*2*^ = 10.5, *P* < .005, *X*^*2*^ = 10.5, *P* < .005, *X*^*2*^ = 6.4, *P* < .04, respectively), in these 4 patients.

Sex, family history of cancer, the number of metastatic lesions, and the effectiveness of adjuvant chemotherapy (as indicated by the percentage of metastatic tumors with necrosis) were not associated with on the total % EPCs/WBC. The level of serum VEGF concentration was higher only in the patients, both those with MSI-H and those with MSS, who had a positive family history of any cancer (*X*^*2*^ = 2.17, *P* < .02), and in patients with the onset of liver metastases earlier than 12 months after the operative treatment of the primary lesion (*X*^*2*^ = 6.81, *P* < .03).

Multi-parametric analysis showed MSI-H status (OR = 9.12, *P* < .01), metastases in both liver lobes (OR = 32.83 *P* < .001) and the presence of simultaneous metastases within and outside the liver (OR = 8.32 *P* < .01), as independent factors associated with higher %CECs/WBC; the serum concentration of angiogenesis VEGF in CRC patients in stage IV of the disease. These associations were found significantly more frequently only in patients with MSI-H colorectal cancer, and not with MSS (*P* < .001). Details are given in Table 3.

**Table 3:**
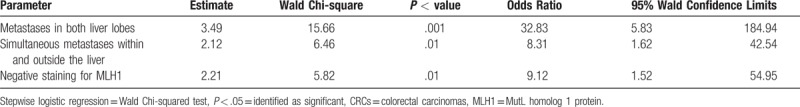
Results of multivariate analysis with stepwise logistic regression of factors indicating for high activity of angiogenesis in CRC patients in stage IV of disease.

## 5 Discussion

The prognostic and predictive values of the MSI-H phenotype in colorectal cancer for creation of metastases are virtually unidentified, as is the influence of tumor genomic integrity on the process of angiogenesis. So the question of whether, and if so, to what extent, the activity of angiogenesis depends on genetic integrity of the tumor is of great importance in the era of personalized cancer treatment.^[41,42]^ The current study revealed significantly higher serum levels of the VEGF protein and higher numbers of circulating EPCs as % of WBC in the cohort of 80 patients with CRC liver metastases in comparison to the cancer-free individuals. Also, in patients with CRC liver metastases who were diagnosed with microsatellite instability (MSI-H) metastases, the number of circulating endothelial cells and the level of cytokines were significantly higher than in those with microsatellite stable (MSS) metastases. The EPCs as percentage of WBC and the VEGF cytokine concentration were mutually correlated in MSI-H patients, whereas in MSS patients and cancer-free individuals they were not. The data clearly indicate that there is a higher propensity for MSI-H colorectal tumors within the liver to exhibit angiogenesis. Thereby, our research confirms earlier suggestions of increased activity of angiogenesis in MSI tumors, as reported by Ellis and Hicklin and Kwon et al.^[5,6]^

The vascular endothelial growth factor (VEGF) is believed to be the strongest stimulator of cancer angiogenesis. The relationship between overexpression of VEGF and the development of liver metastases seems to be mutual as demonstrated in colorectal carcinoma patients by numerous studies.^[4,6–10]^ Kwon et al indicated the preoperative serum VEGF and CRP level as a poor prognostic factor for overall survival in patients with colorectal cancer.^[6]^ Tokunaga et al demonstrated on frozen sections of colon cancer derived from 61 patients who underwent surgical resections, that overexpression of VEGF mRNA is correlated with liver metastasis and poor prognosis.^[10]^ Recently, Samamé Pérez-Vargas et al, as well as Smith and Bhowmick found VEGF/VEGFR pathway synergistic activity with activation of Hepatocyte Growth Factor and the Mesenchymal to Epithelial Transition Factor (HGF/cMET) signaling pathway that contributes to tumor progression and metastases through stimulation of angiogenesis and lymphangiogenesis.^[11,43]^ On the other hand, Yasuhiro Inoue et al, showed suppressed production of angiogenesis growth factors such as VEGF and HGF (hepatic Growth Factor) in frozen sections of the colorectal tumors, and Wendum et al, showed lower expression of VEGF and lower microvessel density (MV) in the paraffin-embedded tumor samples of the MSI-H tumors.^[7,44]^ There is also quite a lot known about the endothelial progenitor cells’ (EPCs) participation in the process of angiogenesis in colorectal tumors. They are an important link in the chain of response to the activity of tumor cytokines, mainly the VEGF-A, which seems to be the most important factor in the recruitment of EPCs to the peripheral blood.^[24–26,45,46]^ Circulating endothelial cell progenitors mobilized by VEGF have been found to promote angiogenesis.^[14,15,18,20]^ High level of EPCs was correlated with advances of the disease and return to normal following antiangiogenic treatment.^[18,26]^ Matsusaka et al and Ronzoni et al have also reported that the high number of EPCs is correlated with poor outcome for patients with metastatic colorectal cancer.^[46,47]^ A number of reports have also explored their potential for monitoring the course of CRC, as well as, a biomarker of cancer disease itself and its response to cancer therapy.^[3,4,12,13,26,48,49]^ However, some believe that the high number of the circulating endothelial cells measured before surgery does not correspond simply to Dukes’ or AJCC stage of carcinoma in CRCs patients, and could not serve as a biomarker predicting the outcome.^[33,35]^ Unfortunately, in the vast majority of research on the proangiogenic cytokines and cells in patients with colorectal carcinoma, as referenced above in the examples, the genomic integrity of tumor are not taken into account in these considerations.

A number of reported studies support the favorable prognosis of patients with MSI-H compared to MSS CRC patients.^[1,30–33]^ It is also suggested that advanced stage MSI-H tumors resemble the early stage of MSS tumors with respect to prognosis, but some data indicate that the prognostic value of MSI is only prominent in stage II cases.^[50,51]^ However, a lot of research notes that more than 40% of patients with MSI-H sporadic CRCs are only diagnosed at the stage IV of disease, and 30% have the BRAF V600E mutation considered to be a significant negative prognostic marker for patients with metastatic CRC.^[37,38,52,53]^ MSI-H sporadic colorectal cancers are usually poorly differentiated and mucinous, with an inflammatory reaction, rich lymphocytic infiltration, and localized more frequently in the proximal colon.^[13,53,54]^ The patients with MSI-H colorectal cancer liver metastases who were selected for the current study frequently had metastases in both liver lobes or synchronous metastases in and outside the liver, as well as infiltration of the liver capsule by the cancer as found by pathologist on explant examination. These were the characteristics found as independent factors associated with higher total % EPCs/WBC and serum concentration of VEGF in the multivariate analysis. However, the most important factor for this association was the MSI status of the metastatic tumor. We assumed, that the loss of MLH1 and/or MSH2 is a sine qua non for microsatellite instability. By the way, this is suggestion of many experts in that field, since loss of PMS2 almost always accompanies loss of MLH1 (rare cases of isolated PMS2 loss are due to PMS2 germline mutations and would not influence our results) and MSH6 is mostly lost with MSH2 (uncommon isolated MSH6 loss is usually in endometrial cancer and also therefore unlikely). Thus it seemed to be redundant to test for all 4 proteins.

In the context of many studies suggesting the role of angiogenesis in the development of both the primary tumor and its metastases,^[13,24–28,41]^ our study supports the view that the metastases are more numerous in those that are MSI-H because these tumors have greater propensity for angiogenesis. Alternatively, it could simply reflect a high burden of disease that drives these and other markers up. The former interpretation is supported by the results of systemic postoperative anti-VEGF therapy with Bevacizumab in 4 of our patients with MSI-H cancers. These patients had significantly fewer EPCs and lower VEGF serum concentration than patients with MSI-H who underwent 5-FU based chemotherapy or anti-EGF therapy applied as a supplementary treatment. The effect of Bevacizumab was very significant in relation to a number of CD309+CD45-stem cells of the endothelium and to a concentration of VEGF, but less significant in relation to number of CD309+CD45+ stem cells from the hematopoietic line. These findings could indicate, although indirectly, a leading role for angiogenesis in metastatic MSI-H related tumors and supports suggestions indicating the resistance of patients with MSI-H colorectal carcinoma to chemotherapy based on 5-FU.^[11,24,27,31]^ This challenges also the view that MSI-H cancers are less aggressive.

Hepatic resection is standard method of treatment of colorectal carcinoma metastases to the liver. The goal is to slow the disease and prolong life. Unfortunately, the majority of patients develop recurrence within several months, even in those with metastases resected with a negative histologic margin of healthy liver tissue.^[55]^ Chemotherapy is usually offered, either before or after surgical treatment, with the aim of prolonging the disease-free course.^[56]^ Size of the primary tumor over 5 cm, positive lymph nodes at primary surgery, the disease-free interval less than 12 months between colorectal resection and the onset of liver metastases, and the presence of more than 1 metastasis are considered as factors predicting poor prognosis.^[37,38,56,57]^ These associations were found significantly more frequently in patients with MSI-H colorectal cancer in the current study. The study proved that MSI patients with colorectal metastases to the liver had significantly more circulating endothelial progenitor cells (EPCs) and higher plasma concentration of VEGF, which in fact reflect increased global level of angiogenesis and thus an unfavorable prognosis for these patients. Thus, these findings indicate the need for determination of genomic integrity in patients with colorectal carcinoma for therapeutic decision-making, at least after resection of liver metastases, and even better after resection of the primary tumor. So, in this respect, our suggestions are in line with the ESMO guidelines for the treatment of patients with metastatic colorectal cancer, although, the recommendation 6 refers to MSI testing for the use of immune checkpoint inhibitors in the treatment of colorectal cancer metastases.^[37,38]^

The question of whether EPCs can serve as a biomarker requires further study and clinical tests. The experience of many research centers indicates that the level of endothelial progenitor cells (EPCs) is essential for tumor growth.^[14–23]^ Recently, Zhu et al^[14]^ revealed that EPCs are mobilized and incorporated into tumor vessels throughout the whole process of hepatocellular carcinoma (HCC) growth and Sun et al demonstrated the role of EPCs in HCC neovascularization.^[23]^ EPCs constitute a relatively small, reaching less than 3%, subpopulation of hematopoietic CD45+ stem cells and a larger, amounting to 35%, subpopulation of vascular endothelium CD45-cells.^[18,20–22]^ Our previous study on patients with an early HCC showed that the number of endothelial progenitor cells (EPCs) in the population of CD34+ stem cells is significantly lower than in patients with an advanced HCC. It also revealed that in all early HCC patients with a relapse within 3 years from radical surgery, the number of EPCs determined at the time of the patients’ qualification for treatment was significantly higher^[22,39]^ These results indicate that the activity of angiogenesis is proportional to how advanced the tumor is. Consequently, the determination of the EPCs in circulation provides an effective way of identifying patients with an advanced cancer.^[15,16,20,39]^ Yet, most importantly, a high number of circulating EPCs indicates the presence, among cancer patients determined on the basis of the clinical classification system, of patients with a tumor of exceptionally high biological activity characteristic of more malignant, more advanced tumors with poor prospects for successful surgical treatment. Determination of the number of circulating EPCs at the time of the qualification of candidates for radical surgical treatment may thus constitute a valuable clue in the proper stratification of patients.^[39]^

Gating the WBC population with the monoclonal antibodies CD34, CD133, CD309 seems to be the most effective method of determining the phenotype of EPCs and allows them to be recognized in the population of WBC. The strategy was established to separate the CD34+CD45+ and CD34+CD45- cell fractions from irrelevant cell populations, as recommended by the International Society of Hematology and Graft Engineering (ISHAGE).^[19–22]^ Following many reports indicating the human endothelial progenitor cells as the primitive progenitors within the hematopoietic and endothelial lines of the stem cells, we added the surface marker CD133 to the original ISHAGE protocol, as the presence of CD133 positivity indicated both the stemness and the hematopoietic lineage of the cells.^[14–23]^ Assuming that the cells positive to CD34, CD133 and CD309 produce the lowest counts in the circulation we also increased the total number of acquired events in the flow cytometer analysis to at least 2,000,000. The immunofluorescence of the cells for CD309 was assessed after identification of CD34 cells within the fraction of cells positive and negative to the CD45 marker. Their counts were added up and expressed as a percentage of EPCS in CD34+ cells population. Flow cytometry pictures indicating the low and high expression of endothelial progenitor cells in the genomic stable and unstable colorectal cancer metastases.

The increased numbers of EPCs potentially indicates the capacity of the tumor to stimulate angiogenesis, however, we are aware that EPCs and VEGF can be derived from other sources, not only from the tumor cells. Nevertheless, we are convinced that the association between tumor genomic instability and increased angiogenesis factors is well founded. We are not necessarily claiming it is cause and effect, but it supports a role for these agents in carcinogenesis. A previous study from our group reported an effective identification of the endothelial stem/progenitor cells in the peripheral circulation which proved to be useful in stratifying of HCC patients for treatment options.^[39,40]^

Although the study results are preliminary, it provides novel information about the biology of genetically unstable sporadic colorectal cancers (MSI-H tumors). Certainly, the study has some important limitations; first it was carried out in the single center; secondly, the analysis concerned a relatively small group of patients. The MSI group consisted of as many as 24 patients. This is a selection bias of our series, but we tried to achieve the test group sufficiently large to perform the statistical analysis. Unfortunately, we were also not able to verify the MSI status of our patients by the repetitive DNA sequences due to organizational limitation. Summarizing, the study revealed that 24 participants with liver metastases from MSI-H primary colorectal carcinomas were characterized by the overexpression of circulating EPCs and VEGF protein level. These findings contrast not only to 30 healthy subjects, but also to 54 participants with the liver metastases from MSS primary colorectal cancers. The overexpression of the angiogenesis promoters could point to a higher propensity of these carcinomas to regrow within the liver after surgical eradication of the metastatic tumor, even though negative margins of tissue were achieved. The analysis of genomic integrity of colorectal carcinoma seems to be, therefore, valuable for designing a combination therapy. However, the question whether the estimate of the specific fractions of circulating EPCs can potentially serve to monitor the course of disease after operation and the possible response to chemotherapy requires further investigation.

## 6 Conclusions

Patients with metastases from MSI-H primary colorectal cancer are characterized by the overexpression of circulating EPCs and VEGF. This supports the likelihood that MSI-H tumors drive angiogenesis. The enumeration of the fractions of EPCs should be considered while therapeutic decision-making process in patients after surgical eradication of the metastases. Determination of genomic integrity in patients with colorectal carcinoma seems to be desirable, at least in the stage IV of disease, in accordance with the principles of personalized medicine.

## Acknowledgments

Authors would like to thank all colleagues for their cooperation.

## Author contributions

**Conceptualization:** Wlodzimierz Otto.

**Data curation:** Janusz Sierdzinski, Justyna Smaga, Maria Krol, Ewa Wolinska.

**Formal analysis:** Wlodzimierz Otto, Janusz Sierdzinski.

**Investigation:** Finlay Macrae, Justyna Smaga.

**Methodology:** Wlodzimierz Otto, Finlay Macrae.

**Project administration:** Wlodzimierz Otto, Janusz Sierdzinski, Maria Krol.

**Resources:** Justyna Smaga, Maria Krol, Ewa Wolinska.

**Software:** Janusz Sierdzinski.

**Supervision:** Wlodzimierz Otto, Finlay Macrae, Krzysztof Zieniewicz.

**Validation:** Finlay Macrae, Janusz Sierdzinski, Maria Krol, Ewa Wolinska, Krzysztof Zieniewicz.

**Visualization:** Janusz Sierdzinski, Justyna Smaga, Maria Krol.

**Writing – original draft:** Wlodzimierz Otto, Finlay Macrae, Krzysztof Zieniewicz.

**Writing – review & editing:** Finlay Macrae, Krzysztof Zieniewicz.
